# Auditory Mismatch Negativity in Response to Changes of Counter-Balanced Interaural Time and Level Differences

**DOI:** 10.3389/fnins.2017.00387

**Published:** 2017-07-06

**Authors:** Christian F. Altmann, Ryuhei Ueda, Shigeto Furukawa, Makio Kashino, Tatsuya Mima, Hidenao Fukuyama

**Affiliations:** ^1^Human Brain Research Center, Graduate School of Medicine, Kyoto UniversityKyoto, Japan; ^2^Department of Psychology, Graduate School of Letters, Kyoto UniversityKyoto, Japan; ^3^NTT Communication Science Laboratories, NTT CorporationAtsugi, Japan; ^4^Graduate School of Core Ethics and Frontier Science, Ritsumeikan UniversityKyoto, Japan

**Keywords:** auditory evoked potentials, cue integration, electroencephalography, mismatch negativity, sound localization, spatial hearing

## Abstract

Interaural time differences (ITD) and interaural level differences (ILD) both signal horizontal sound source location. To achieve a unified percept of our acoustic environment, these two cues require integration. In the present study, we tested this integration of ITD and ILD with electroencephalography (EEG) by measuring the mismatch negativity (MMN). The MMN can arise in response to spatial changes and is at least partly generated in auditory cortex. In our study, we aimed at testing for an MMN in response to stimuli with counter-balanced ITD/ILD cues. To this end, we employed a roving oddball paradigm with alternating sound sequences in two types of blocks: (a) lateralized stimuli with congruently combined ITD/ILD cues and (b) midline stimuli created by counter-balanced, incongruently combined ITD/ILD cues. We observed a significant MMN peaking at about 112–128 ms after change onset for the congruent ITD/ILD cues, for both lower (0.5 kHz) and higher carrier frequency (4 kHz). More importantly, we also observed significant MMN peaking at about 129 ms for incongruently combined ITD/ILD cues, but this effect was only detectable in the lower frequency range (0.5 kHz). There were no significant differences of the MMN responses for the two types of cue combinations (congruent/incongruent). These results suggest that—at least in the lower frequency ranges (0.5 kHz)—ITD and ILD are processed independently at the level of the MMN in auditory cortex.

## Introduction

How does the brain integrate information from different modalities or within a single modality? Solving this question is a necessary step to understand perception, and it would also advance us on the path to understand general brain function. In the auditory field, binaural sound localization in the horizontal plane provides a remarkable model for cue integration: two cues, interaural time (ITD) and level differences (ILD), define horizontal sound source position (left/right), and for a non-contradictory perception of our environment they have to be aligned with each other. The duplex-theory of sound localization states that ITD and ILD dominate in different frequency ranges (Strutt, 1907), and later studies have provided evidence that the binaural processing pathway exhibits accordingly an anatomical separation early in the brain stem (for a review see, e.g., Grothe et al., 2010). In particular, the early low frequency ITD pathway is mainly based on processing in the medial superior olive, while the early high frequency ILD pathway involves the lateral superior olive in the brain stem.

However, our everyday experience tells us that at some point along the binaural pathways, ITD and ILD are at least partly integrated: usually, we do not perceive a world of ITD and one of ILD sound sources. This is demonstrated with psychophysics, for example, with the existence of ITD/ILD cue trading (Shaxby and Gage, 1932; Hafter and Jeffress, 1968; Furukawa, 2008): a right lateralized sound source, for instance, defined by ITD can be counter-balanced toward the midline by adding an opposing ILD cue, and vice versa. In a previous study, we employed electroencephalography (EEG) to test whether ITD/ILD integration is reflected at the level of the so-called mismatch negativity (MMN; Altmann et al., 2014). The MMN arises in response to changes of an acoustic stimulus (e.g., sound source location changes) with generators in auditory cortex (e.g., Deouell et al., 2006). In our study, we observed a significant MMN in response to a change from a midline stimulus to a stimulus with counter-balanced ITD and ILD, perceptually located at midline (Altmann et al., 2014). This indicated independent processing in the two frequency ranges (500 and 4,000 Hz) that we tested. However, we also included stimuli in which only one of the cues, ITD or ILD, was non-zero to test for linearity of cue combinations and found for the lower frequency range (500 Hz), that the sum of the MMN in response to changes of only one cue, ITD or ILD, exceeded significantly the MMN in response to the combined stimulus. Thus, our study provided evidence for both independent processing, but also signs of integrated processing. This is to some extent in agreement with another recent EEG study that has described integrated ITD/ILD processing with some retention of independent information for late auditory evoked potentials (Edmonds and Krumbholz, 2014).

The interpretation of our previous findings as evidence for independent ITD/ILD processing was based on the observation of significant MMN responses to counter-balanced ITD/ILD cues (Altmann et al., 2014). However, an alternative explanation for the occurrence of an MMN might have been that it was mainly elicited by a transition from a clear midline sound stimulus to an unnatural ITD/ILD combination. Previous psychophysical studies have suggested that naturally occurring ITD/ILD combinations can lead to very different impressions compared to contradictory parameters (Gaik, 1993). Specifically, previous studies have suggested that some unnatural cue combinations can result in multiple auditory images (Sayers, 1964; Hafter and Jeffress, 1968).

In the present study, we aimed at addressing the question whether MMN to counter-balanced ITD/ILD can occur even in the absence of a transition from a midline intracranial image to the unnatural cue combination. To this end, we employed a roving-oddball paradigm in which we presented sequences of repeated sound stimuli with either congruent or incongruent ITD/ILD cues. MMN was evoked by changing the sign of the ITD and ILD cues which, in case of congruently combined ITD/ILD, led to a change in perceived sound lateralization (left → right and vice versa). In case of incongruently combined ITD/ILD, a sign change did not result in a perceived change of lateralization; instead, the sound source continued to remain in the midline. This was different to our previous study (Altmann et al., 2014) in which changes always entailed a transition from zero to non-zero ITD/ILD, whereas in the present study all stimuli entailed non-zero ITD/ILD. For our current study, we hypothesized that significant MMN in response to both congruent and incongruent ITD/ILD changes would suggest independent processing of these two cues at the level of the MMN. In contrast, a significant difference between the MMN for congruent and incongruent ITD/ILD changes would point to an integration of these two cues. We tested in two different frequency ranges (500 and 4,000 Hz) to address the differences in integrative processes in these frequency ranges described in a previous behavioral study (Furukawa, 2008).

## Materials and methods

### Participants

This EEG experiment was conducted on 24 healthy, normal hearing volunteers. One subject was excluded due to ocular artifacts that compromised more than 50% of the data epochs. The analyzed sample consisted of 12 males and 11 females with an average age of 23.9 years (range: 21–38). Twenty-one participants were right-handed as determined by self-report, one participant was originally left-handed but switched to right-handedness in childhood, and one participant was left-handed. All participants were informed of the aims and risks of the experiment and gave written informed consent. The experiments were performed in accordance with the ethical standards laid down in the declaration of Helsinki of 1964 and the guidelines approved by the local ethics committee of the Graduate School of Medicine and Faculty of Medicine, Kyoto University.

### Experimental stimuli and apparatus

**Figure 1:**
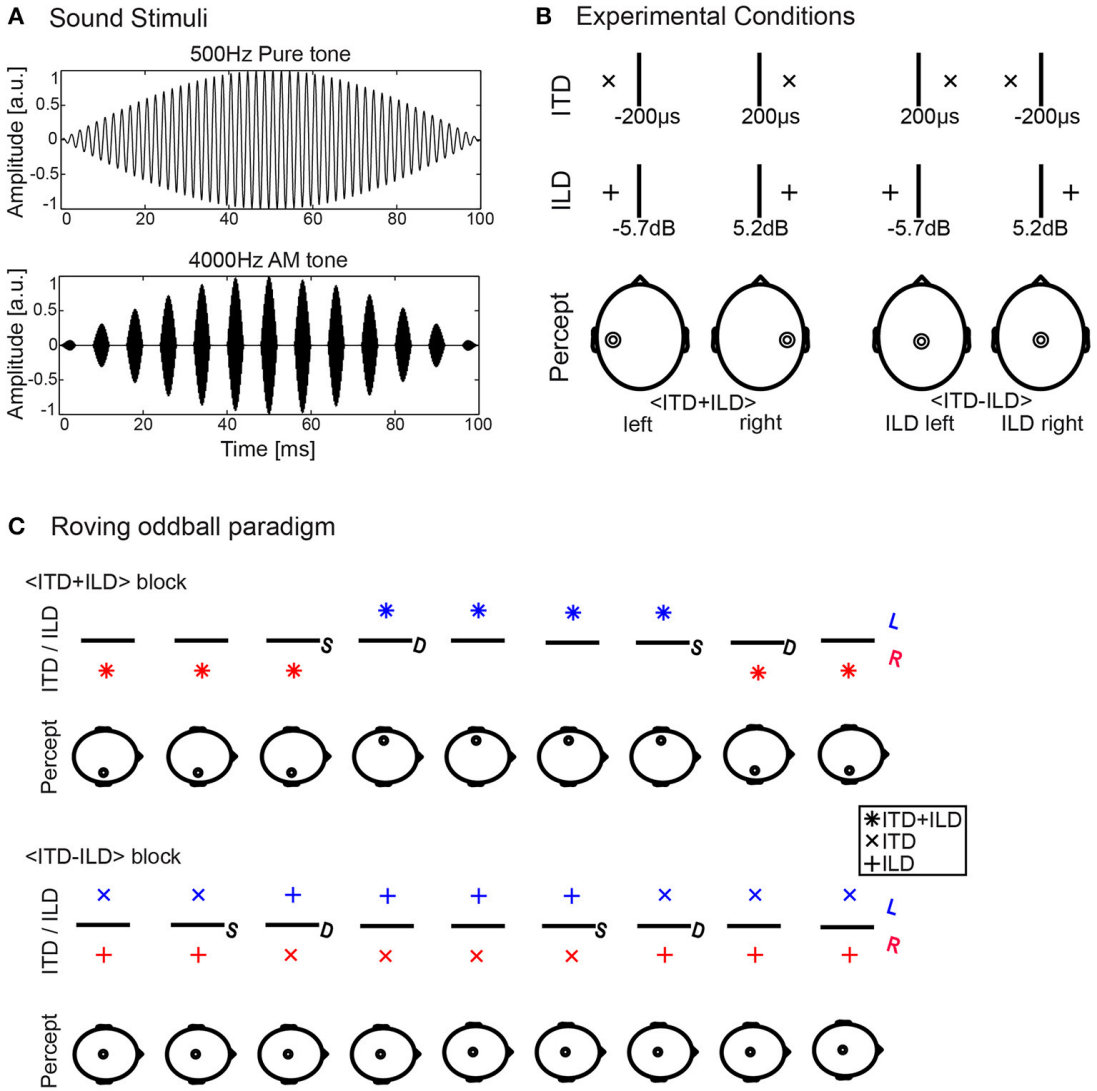
Experimental methods. **(A)** Sound stimuli consisted either of 500 Hz pure tones (PT500, upper oscillogram) or 4,000 Hz tones, amplitude-modulated by a half-wave rectified 125 Hz sinusoid (AM4000, lower oscillogram). **(B)** Experimental conditions. The top row shows the ITD parameters, the center row the ILD parameters and the bottom row the hypothesized percept resulting from the combination of ITD and ILD in the tested conditions. The values show the parameter averages determined for the PT500 stimulus (*n* = 24). **(C)** Stimulus sequences. We employed a roving-oddball paradigm to elicit the MMN. The stimulus series of congruent (<ITD+ILD>, upper row) and incongruent (<ITD-ILD>, lower row) standards and deviants were presented block-wise. L, Left; R, Right; S, Standard; D, Deviant.

The sound stimuli, depicted in Figure 1A, had a sampling rate of 96 kHz and were similar to those used in our previous study (Altmann et al., 2014). They consisted of either pure tones with a carrier frequency of 500 Hz (PT500) or tones with a carrier frequency of 4 kHz, amplitude-modulated by a half-wave rectified 125 Hz sinusoid (AM4000). The AM4000 stimulus was a so-called “transposed stimulus” that allows for testing ITD sensitivity with a high-frequency carrier signal (van de Par and Kohlrausch, 1997; Bernstein, 2001; Bernstein and Trahiotis, 2002; Furukawa, 2008). The modulator of the AM4000 stimulus was low-pass filtered with a cutoff frequency of 2 kHz to restrict the energy of the resulting stimulus to the ±2 kHz range around the carrier frequency. All sound stimuli had a duration of 100 ms and were shaped by a 50 ms rising and falling diotic cosine ramp. This long ramp was employed to reduce the saliency of the sound onset and emphasizing ongoing ITD/ILD cues (Furukawa, 2008). Previous studies have shown dominance of either onset or ongoing ITD cues depending on the frequency range of the stimuli (Buell et al., 1991; Brown and Stecker, 2010). Sound pressure was adjusted to 45 dB above individual sensation level (SL) determined for the two types of sounds separately. Simulated in-head lateralization was induced by adding ITD and ILD cues. The ILD was implemented by increasing the level in one ear and decreasing it in the other relative to the center level (45 dB SL), so that the average across the two ears on a decibel scale was the center level.

Two types of ITD/ILD combinations were created for each frequency range (see Figure 1B): (a) stimuli with negative/positive ILD and congruent ITD to elicit the perception of a left/right-lateralized intracranial sound image: <ITD+ILD>, and (b) stimuli with negative/positive ILD and incongruent positive/negative ITD to elicit the perception of a central intracranial sound image: <ITD-ILD>. Similar to our previous MMN study (Altmann et al., 2014), for PT500, the ITD was always fixed at either −200 or +200 μs and the ITD for the AM4000 was determined by matching the perceived lateralization of the AM4000 to that of the PT500. This matching led on average to ITDs of −469 ± 217 (SD; range: −840 to −185) and +440 ± 213 (SD; range: +160 to −985) μs. The ILDs for congruent and incongruent combinations were determined by a psychophysical ITD/ILD cue trading procedure similar to our previous study (Altmann et al., 2014). Based on the individual results of this procedure we used in the EEG experiment average ILDs of 5.2 ± 2.5 (SD; range: 2.5–13.5) dB and −5.7 ± 2.2 (SD; range: −10.5 to −2.5) dB for PT500. For AM4000, we used 3.4 ± 2.0 (SD; range: 0.5 to −9) dB and −4.2 ± 2.4 (SD; range: −0.5 to −9.5) dB.

Sounds were delivered via Etymotic Research ER4 in-ear-headphones (Etymotic Research Inc., Elk Grove Village, IL, USA) connected to a USB audio interface (M-Audio Fast-Track Pro, M-Audio Inc., Irwindale, CA, USA). Stimulus presentation was controlled by a PC using the Psychophysics Toolbox (Brainard, 1997; Pelli, 1997; Kleiner et al., 2007), running in a Matlab environment (R2010b, The Mathworks Inc., Natick, MA, USA). During all parts of the experiment, subjects were seated in a single-walled acoustic booth (AT-66, RION Co., Ltd., Kokubunji, Japan).

### Procedure

For stimulus generation, we first determined the diotic sensation levels for each stimulus type (PT500, AM4000) separately as the 50% detection threshold with a weighted up-down method (Kaernbach, 1991). This was followed by matching the lateralization of the ITD for the PT500 and AM4000 sound stimuli and then by an ITD/ILD cue trading procedure, similar to our previous MMN study (Altmann et al., 2014).

During the EEG experiment, subjects watched a self-chosen subtitled movie on a computer screen placed outside the acoustic booth through a window. They were instructed to keep their eyes fixated on the screen, not to close their eyes and keep ocular and head motion to a minimum. The EEG experiment consisted of four experimental blocks of stimulus presentation, one for PT500 with congruent ITD/ILD combinations (<ITD+ILD>), one for PT500 with incongruent ITD/ILD combinations (<ITD-ILD>), one for AM4000 with congruent ITD/ILD combinations (<ITD+ILD>), and one for AM4000 with incongruent ITD/ILD combinations (<ITD-ILD>). The presentation order of these four different blocks was balanced across subjects. Stimuli were presented according to a roving oddball paradigm in which sequences of repeated stimuli were alternated, similar to previous EEG studies (e.g., Altmann et al., 2007). In a traditional oddball design, rare deviants interrupt sequences of standard sound stimuli. In contrast, in the roving oddball paradigm, deviants are defined as the first stimuli after a repeated presentation of another stimulus and serve then—after repeated presentation—themselves as standard (see Figure 1C). The last stimulus in such a sequence of repeated sounds was defined as standard, and the MMN was calculated as the difference waveform between the deviant and standard event-related potentials (ERPs). In each repetition sequence, 3–10 (average 6.5) instances of a stimulus were repeated, stimulus-onset-asynchrony (SOA) was 0.6 s and each block consisted of 129 sequences, i.e., on average about 838.5 stimulus presentations (block duration: about 8 min 23 s). This resulted in 128 standards and deviants per condition and participant (pooled over left-to-right and right-to-left changes). In addition to this MMN experiment, subjects participated in an unrelated EEG experiment that consisted of four experimental blocks with a duration of about 9 min each. The order of experiments was balanced across subjects, to avoid systematic effects of fatigue.

### EEG data acquisition and analysis

The continuous EEG was sampled at 500 Hz using 32 Ag/AgCl electrodes with a QuickAmp amplifier (Brain Products GmbH, Munich, Germany). Electrodes were placed on an electrode cap (EasyCap GmbH, Herrsching, Germany) with a cap size determined by the subjects' head circumference (54, 56, 58, or 60 cm). The electrode positions were based on the International 10–10 system. The ground electrode was placed on the forehead (position: Fpz) and the EEG signal was recorded with an average reference, later re-referenced to the average of left and right mastoid electrodes to maximize the MMN amplitude, similar to previous studies (Schröger, 1996). Electrode impedance was generally kept below 5 kΩ (except two participants: <10 kΩ). EEG data were analyzed with the BESA software package (BESA GmbH, Gräfelfing, Germany) and custom-written Matlab software. The raw EEG was high-pass filtered with a cut-off frequency of 0.1 Hz. The event-related epochs were defined from −100 to 400 ms after stimulus onset. Artifactual epochs were discarded based on a thresholding procedure which removed epochs with a peak-to-peak amplitude exceeding 120 μV and a slew rate exceeding 75 μV/ms. In addition, visual inspection of the raw signal was performed to further exclude ocular motion and muscle artifacts. This resulted in on average 103.4 (81%) and 102.5 (80%) trials per condition for the PT500 and AM4000 runs, respectively. The event-related averages were low-pass filtered with a cut-off frequency of 25 Hz.

We statistically tested for the occurrence of an MMN with a cluster-based permutation analysis as implemented in the FieldTrip toolbox (http://fieldtrip.fcdonders.nl/; Maris, 2004; Maris and Oostenveld, 2007). This approach addresses the problem of multiple comparisons across time and electrode positions. As we had a-priori information on the MMN elicited by similar stimuli from our previous study (Altmann et al., 2014), we compared deviants and standards using a one-tailed Student's *t*-test in the MMN time window between 110 and 170 ms after stimulus onset. Clusters were formed of significant electrodes (minimum: 2) and time-points that showed a significant difference between deviant and standard (*p* < 0.05). As a test-statistic, the sum of *t*-values across a cluster was compared to the distribution of maximum cluster sums of *t*-values derived from a randomization procedure (2,000 random permutations of the observed data). Differences were reported as statistically significant when the cluster *p*-value was below a critical α-value of 0.05, corrected for multiple comparisons.

Additionally, in order to compare between congruent and incongruent MMNs, we estimated the MMN peak amplitudes and latencies at the Fz electrode which has been described to show maximal MMN amplitude in previous studies (e.g., Schröger, 1996; Altmann et al., 2014). We determined the MMN peak latency and amplitude as the most negative peak in the ERP difference waveform between 100 and 170 ms after stimulus onset employing a jackknife procedure (Kiesel et al., 2008). The jackknife approach estimates parameters based on the grand average waveforms of n sub-samples of n-1 participants (n: number of participants). Calculated standard error of the mean values and test statistics (such as *F*-values) require adjustment as suggested for the use of jackknife methods (Miller et al., 1998; Ulrich and Miller, 2001; Kiesel et al., 2008). For the comparison of MMN peak amplitudes and latencies, we conducted two two-way repeated measurements ANOVAs to test for main effects of and an interaction between the factors Stimulus Type (PT500, AM4000) and Condition (congruent, incongruent ITD/ILD).

### Behavioral control experiment

Behaviorally, ITD/ILD cue trading has been reported to be incomplete in previous studies (Hafter and Carrier, 1972; Ruotolo et al., 1979). This means that listeners can sometimes discriminate dichotic stimuli with counter-balanced ITD/ILD from diotic stimuli. It is possible that MMN elicited for counter-balanced ITD/ILD is based on this incompleteness. To estimate detectability of changes in the different experimental blocks, we conducted an additional behavioral control experiment with six volunteers. Of these, five have also participated in the EEG experiment (with at least 2.5 years between EEG experiment and behavioral control), one volunteer, a 23 years old female, was added to this sample.

We employed the same sound stimulation as in the original MMN experiment, but asked volunteers to push a keyboard button whenever they perceived an acoustic change. The stimulation sequences entailed for each of four experimental blocks 128 signal (change) trials embedded in on average 710.5 no-signal trials. No visual stimulus or movie was presented during this experiment. We estimated hits as those responses occurring within 1.2 s after deviant onset; false alarms were responses given outside this time-window. We calculated individual d'-values as a measure for sensitivity and their 95% confidence intervals as described in Macmillan and Creelman (2005). Significant sensitivity of an individual listener for an acoustic change was defined when all values in this 95% confidence interval of the d'-value were larger than 0.

## Results

### EEG experiment

The cue trading procedure resulted in average ILDs of 5.2 ± 2.5 (SD) dB and −5.7 ± 2.2 (SD) dB for PT500; and 3.4 ± 2.0 (SD) dB and −4.2 ± 2.4 (SD) dB for AM4000.

**Figure 2:**
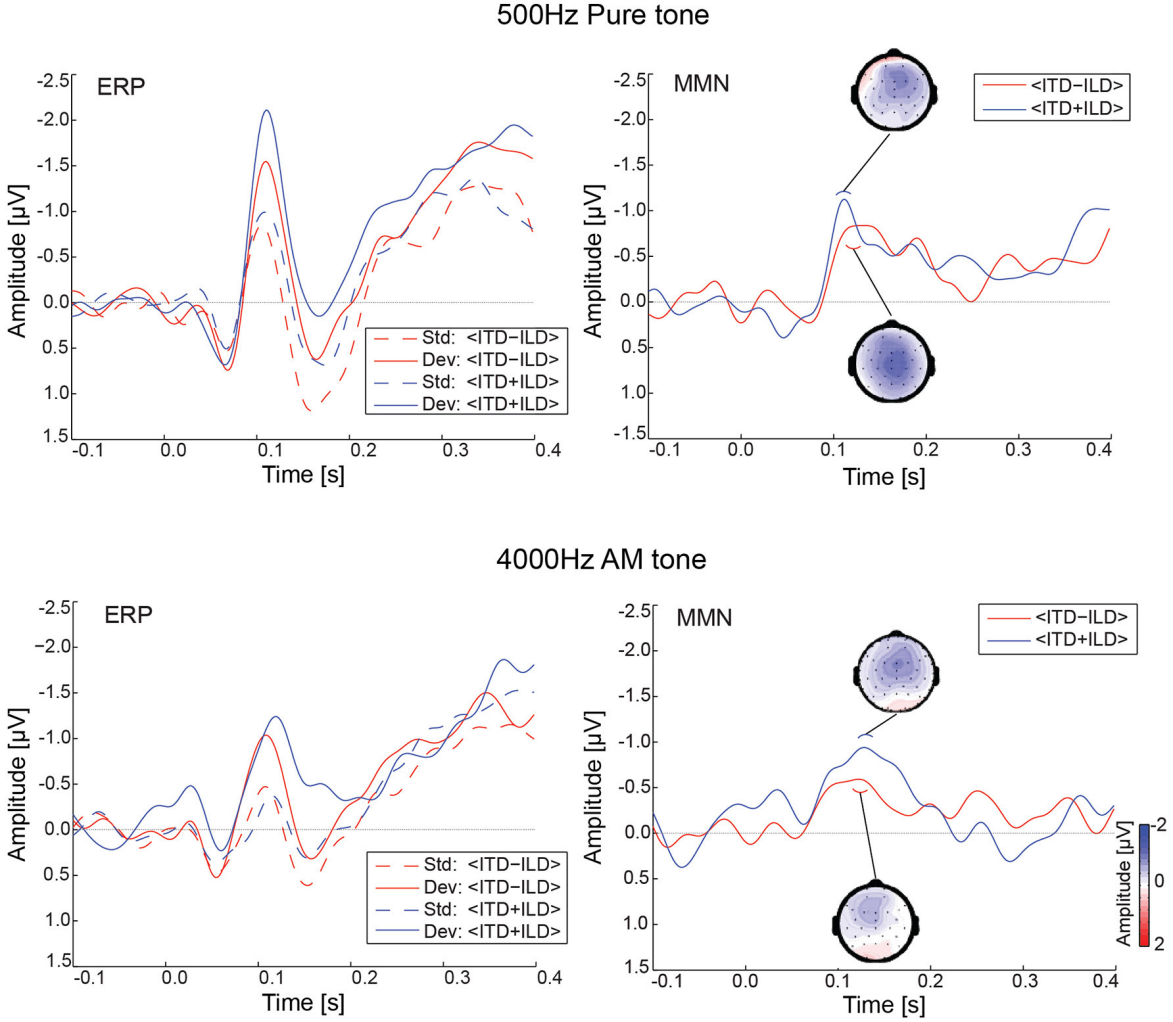
Evoked potentials at electrode Fz (*n* = 23). ERP time-courses are shown on the left side and the corresponding MMN (deviant-standard) waveforms are shown on the right for PT500 **(Top)** and AM4000 **(Bottom)**. The small inlay graphs show the MMN topographies averaged across a 20 ms time window centered on the MMN peak latency in the respective condition. The color-bar in the right lower graph provides the amplitude reference for the MMN topographies. Std, Standard; Dev, Deviant.

Figure 2 depicts the ERPs and MMN waveforms for the PT500 and the AM4000 sound stimuli. For PT500, a cluster-based permutation test revealed a significant MMN for the congruent (<ITD+ILD>) deviant compared to standard (*p* = 0.0025), and for the incongruent (<ITD-ILD>) deviant compared to standard (*p* = 0.0055). In case of the AM4000, only the congruent (<ITD+ILD>) deviant resulted in a significant MMN (*p* = 0.0015), but the incongruent (<ITD-ILD>) deviant did not.

Table 1 shows the MMN peak amplitudes and latencies for the PT500 and AM4000 deviants estimated at electrode Fz. We entered this data into a two-way repeated measurements ANOVA with the factors Stimulus Type (PT500 and AM4000) and Condition (<ITD+ILD>, <ITD-ILD>). Significant effects were neither observed for the MMN amplitudes [all *F*-values < 1, except for the main effect of Condition: *F*_(1, 22)_ = 1.96; *p* = 0.18] nor for the latencies (all *F*-values < 1).

**Table 1:** MMN mean peak amplitudes [μV] ± SEM and latencies [ms] ± SEM for electrode Fz (SEM: adjusted standard error of the mean).

	**PT500** **<ITD+ILD>**	**PT500** **<ITD-ILD>**	**AM4000** **<ITD+ILD>**	**AM4000** **<ITD-ILD>**
Amplitude [μV]	−1.13 ± 0.24	−0.85 ± 0.25	−0.94 ± 0.25	−0.60 ± 0.25
Latency [ms]	112 ± 4	129 ± 39	128 ± 9	119 ± 42

### Behavioral control experiment

We tested six participants whether they could detect any changes of the sound stimuli when presented in a similar manner as in the EEG experiment. The results of the change detection task are shown in Table 2. We found that for the PT500 stimulus, 5/6 participants showed better detection (d') in the congruent (<ITD+ILD>) compared to the incongruent (<ITD-ILD>) block. For the AM4000 stimulus, 6/6 showed better detection (d') for the congruent (<ITD+ILD>) compared to the incongruent (<ITD-ILD>) cue combination. In addition, in the incongruent condition (<ITD-ILD>) d'-values were larger for the PT500 compared to the AM4000 stimulus in 6/6 listeners. When calculating the 95% confidence interval for d' of individual listeners, we found significant (i.e., d' above 0) sensitivity in 6/6 for the congruent (<ITD+ILD>) conditions, for 4/6 for the PT500 incongruent (<ITD-ILD>), but only for 2/6 participants for the AM4000 incongruent (<ITD-ILD>) condition. Thus, this behavioral control experiment indicates that ITD/ILD cue trading can indeed be incomplete, i.e., acoustic changes can be detected even for counter-balanced ITD/ILD changes, in particular for low-frequency stimuli.

**Table 2:** Behavioral change detection for six participants.

	**PT500** **<ITD+ILD>**	**PT500** **<ITD-ILD>**	**AM4000** **<ITD+ILD>**	**AM4000** **<ITD-ILD>**
d' [range]	3.4 [2.5; 4.1]	1.5 [−0.1; 3.8]	2.9 [1.2; 4.6]	0.1 [−0.9; 1.3]
Hits (%) [range]	93.0 [78.1; 99.2]	49.6 [10.9; 96.1]	81.5 [39.1; 98.4]	21.5 [4.0; 49.2]
False alarms (%) [range]	4.4 [2.3; 7.8]	9.9 [2.3; 30.5]	8.5 [0.8; 25.8]	16.4 [4.7; 48.4]

*The table depicts mean d' values, hits, and false alarms rates in percent and the range of those values in square brackets*.

## Discussion

In this study, we tested whether ITD and ILD cues are integrated at the level of the MMN in low and high frequency ranges. To this end, we employed cue-trading of ITD and ILD to generate stimuli that differed in their ITD and ILD cues, but were similar in that they elicited the percept of midline sound source locations. We detected significant MMN for the low (0.5 kHz), but not the high (transposed tone with 4 kHz carrier frequency) frequency range, suggesting independent processing of ITD and ILD in particular for the lower frequencies. A behavioral control experiment in a small sample suggested that listeners are to some extent sensitive to deviants in the incongruent blocks in particular when stimulated with the low-frequency sound (PT500). The overall pattern of psychophysical sensitivity to counter-balanced ITD/ILD thus resembled the MMN results.

ITD/ILD cue cancelation can be incomplete, i.e., even in case that ITD and ILD are optimally counter-balanced, it might still be possible to distinguish the resulting dichotic from a diotic signal (Hafter and Carrier, 1972; Ruotolo et al., 1979). In the context of our current study, this means that incomplete cue trading in particular in the low-frequency range might have contributed to the generation of the MMN in response to deviants in the blocks with incongruently combined ITD/ILD cues.

The finding of an MMN in response to changes of counter-balanced ITD/ILD cues, even when the percept of a midline location remains the same, is in line with our previous MMN study that revealed partly, but not complete, cue independence (Altmann et al., 2014). In this previous study evidence for some cue integration in the lower frequency range was found in the form of sub-additivity of MMN to combined ITD/ILD compared to changes of only one localization cue (either ITD or ILD). This was not in agreement with an earlier MMN study that has described linear additivity for a low-frequency pure tone, but sub-additivity for a complex tone, overall suggesting at least partly independent processing of ITD/ILD (Schröger, 1996). A recent study with a different paradigm, employing changes of ITD/ILD and their combination after presentation of an adaptor, has provided evidence that points toward an integrated ITD/ILD code with the possibility of some residual independent coding (Edmonds and Krumbholz, 2014). Similarly, a recent magnetoencephalography (MEG) study has observed location-specific adaptation of the N1 response of auditory cortex across ITD/ILD cues (Salminen et al., 2015). The authors have interpreted this as an indication of integrated ITD/ILD processing, but given that adaptation effects in their study were larger within compared to across cues, they suggest that neural populations with separate selectivity are likely to exist in auditory cortex.

While the MMN in response to incongruently combined ITD and ILD cues was statistically significant in the lower frequency range, we could not detect a significant MMN in the higher frequency range. Strong indication of independent processing for the low and less in the high frequency range is reminiscent of behavioral findings of stronger interaction in the high frequency range with similar stimuli (Furukawa, 2008). Nevertheless, the non-significance of MMN amplitude differences between congruent and incongruent cue combinations precludes us from confidently inferring integrated ITD/ILD processing for the higher frequency range at the level of the MMN. Of course, our sample size of 23 participants used in this study would have only allowed us to detect large effects with adequate power (Button et al., 2013). Nonetheless, the present study's sample size was comparable to our previous study (*n* = 19; Altmann et al., 2014) and comparable to current standards in MMN studies. Thus, it is possible that the MMN effect size for higher frequencies is in a range that requires larger sample sizes to be detected reliably. This is corroborated by the observation that—while statistically significant—the MMN to the incongruently combined ITD/ILD for the AM4000 tone was also not very pronounced in our previous study (Altmann et al., 2014). In addition, the MMN amplitudes in the current study were only about 50–80% (PT500) and about 90% (AM4000) of those observed in our previous MMN study, which possibly has led to a lower signal-to-noise ratio in the present study. This reduction in absolute MMN amplitude is likely to be an effect of increased deviant probability (50% within a block in the current study vs. 1.67% in the previous study). MMN amplitude has been shown to be reduced as a function of increasing deviant probability in previous MMN studies in humans (Javitt et al., 1998; Sabri and Campbell, 2001) and, for example, cats (Pincze et al., 2002).

Another factor that might have made it difficult to obtain a significant MMN in the higher frequency range in this compared to our previous MMN study might have been the contribution of the “naturalness” of ITD/ILD cue combination. In particular, in our previous study (Altmann et al., 2014) the incongruent ITD/ILD deviants entailed a change from a midline stimulus with naturally occurring ITD/ILD combinations (ITD: 0 μs; ILD: 0 dB) to an unusual combination of counter-balanced ITD/ILD (e.g., ITD: 200 μs; ILD: −5.4 dB). Psychophysical studies have described that such “unnatural” cue combinations can broaden or even split the intracranial image (Sayers, 1964; Hafter and Jeffress, 1968; Gaik, 1993; Furukawa, 2012). Thus, while in our previous study a switch from a single midline image to a broadened or multiple intracranial images could have contributed to the MMN, this effect would have been diminished in the current study. The extent of this effect of naturalness is hard to estimate by comparing across studies, though. Future studies that parametrically manipulate the ITD/ILD cue combinations in regard to their naturalness could possibly speak to this issue.

Conversely, the occurrence of two split intracranial images during presentation of traded ITD/ILD cues might have contributed to the generation of the significant MMN for the low frequency range in this study. Specifically, Hafter and Jeffress (1968) have described that listeners in an ITD/ILD cue trading task may sometimes report two intracranial sound images. With practice, they can learn to adjust the trading parameters to them separately, with one of the images more influenced by time, the other one more influenced by intensity differences, or a combination of time and intensity (see Ruotolo et al., 1979). It cannot be excluded that in the present study, the occurrence of two intracranial images has partly influenced MMN responses: possibly, listeners have counterbalanced only one of the images. The residual intracranial image might have undergone shifts from left to right and vice versa even for the incongruent deviants, causing a MMN response. This illustrates that, while cue trading is an impressive demonstration of ITD/ILD integration, it has its limits at the perceptual level which are likely to be reflected neurophysiologically.

## Conclusions

In sum, our data suggest independent processing of counterbalanced ITD and ILD at the level of the MMN in auditory cortex with a peak latency of about 130 ms after a stimulus change. This was detectable only for a low frequency range (500 Hz pure tone). Employing a roving oddball paradigm allowed us to verify and extend the findings of our previous study (Altmann et al., 2014), in that we now have evidence that swapping the direction of counter-balanced ITD/ILD cues for a sound can elicit a significant MMN while preserving the percept of a midline intracranial image.

## Author contributions

CA, SF, MK, TM, and HF conceived and designed this study. CA and RU acquired and analyzed the data. CA and SF interpreted the data and wrote the manuscript. All authors revised the manuscript critically and approved publication in its final form.

### Conflict of interest statement

The authors declare that the research was conducted in the absence of any commercial or financial relationships that could be construed as a potential conflict of interest.

## Abbreviations

EEG: electroencephalography
ERP: event-related potential
ILD: interaural level difference
ITD: interaural time difference
MEG: magnetoencephalography
MMN: mismatch negativity
SL: sensation level
2AFC: two-alternative-forced-choice.
